# Supplementary material for: Characterization of Volatile Organic Compounds in Kiwiberries (Actinidia arguta) Exposed to High Hydrostatic Pressure Processing by HS-SPME/GC-MS
Source: Molecules. 2022 Sep 12;27(18):5914. doi: 10.3390/molecules27185914 (PMC9502335; doi:10.3390/molecules27185914)
Supplement: Supplementary file 1 [file molecules-27-05914-s001.zip › molecules-1884671-supplementary.pdf]

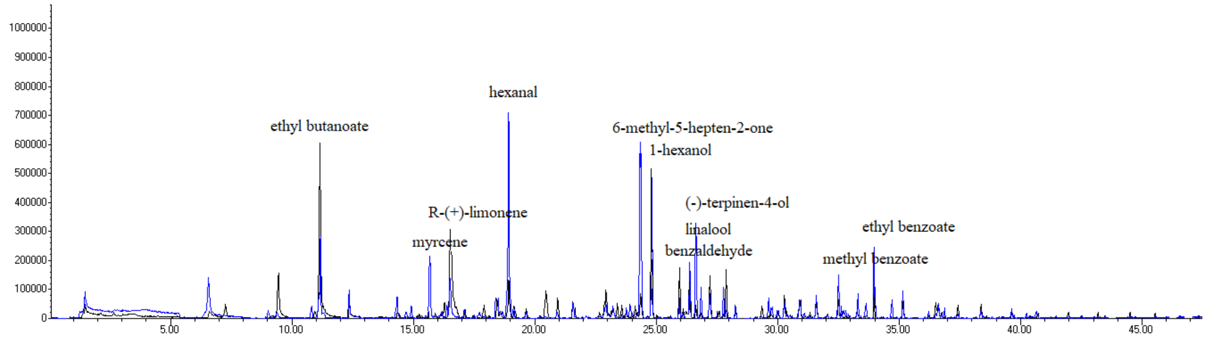

Figure S1. Chromatogram of GC-MS analysis of kiwiberry *cv* Geneva (the untreated sample- blue and HPP-treated 450MPa/5 min- black line). ethyl butanoate; myrcene; (R)-(+)-limonene; hexanal; terpinolene; 6-methyl-5-hepten-2-one; 1-hexanol; benzaldehyde; linalool; (-)-terpinen-4-ol; methyl benzoate; ethyl benzoate

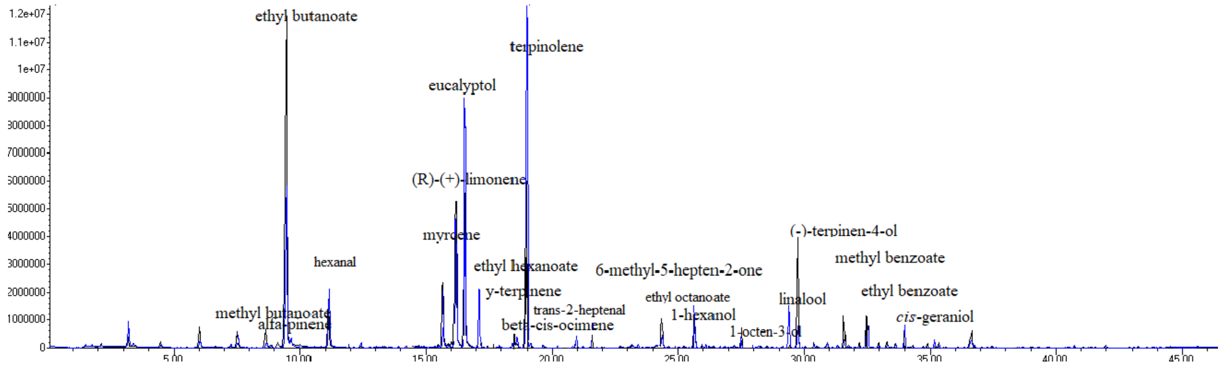

Figure S2. Chromatogram of GC-MS analysis of kiwiberry *cv* Weiki (the untreated sample- blue and HPP-treated 450MPa/5 min- black line). methyl butanoate; α-pinene; ethyl butanoate; hexanal; myrcene; (R)-(+)-limonene; eucalyptol; ethyl hexanoate; γ-terpinene; β-*cis*-ocimene; terpinolene; *trans*-2-heptenal; 6-methyl-5-hepten-2-one; 1-hexanol; ethyl octanoate; 1-octen-3-ol; linalool; (-)-terpinen-4-ol; methyl benzoate; ethyl benzoate; *cis*-geraniol
